# Supplementary material for: Effect of Mitotane on Male Gonadal Function
Source: Cancers (Basel). 2023 Jun 18;15(12):3234. doi: 10.3390/cancers15123234 (PMC10296642; doi:10.3390/cancers15123234)
Supplement: Supplementary file 1 [file cancers-15-03234-s001.zip › cancers-2396992-supplementary.pdf]

Supplementary data

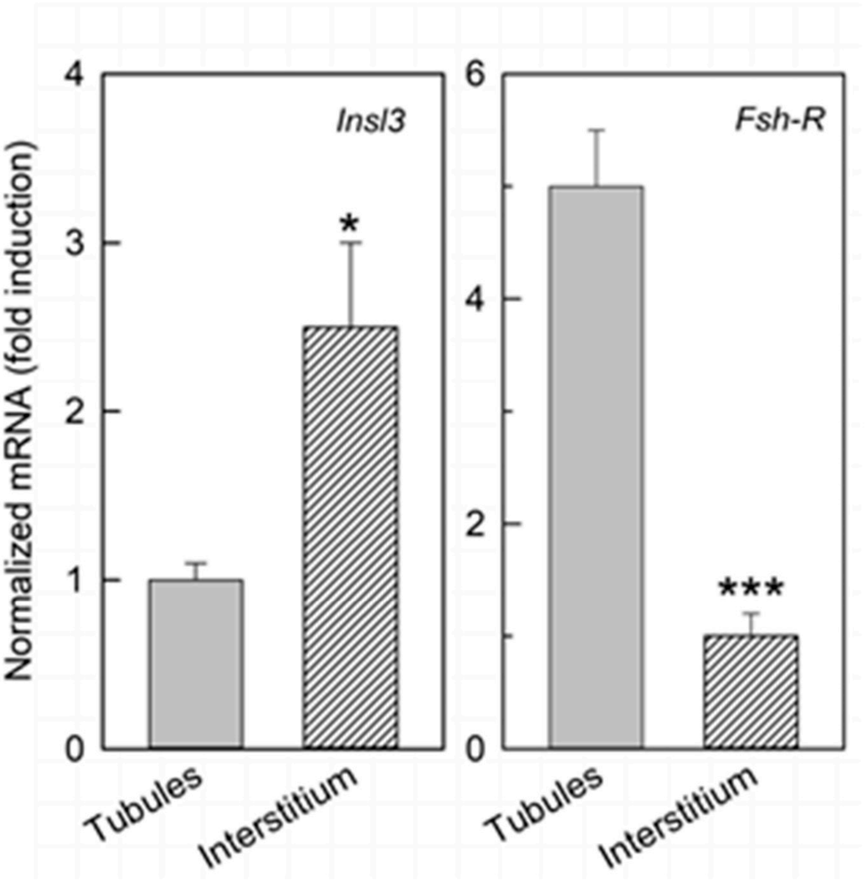

Figure S1. *Fsh-R* and *Insl3* expression in seminiferous tubules and interstitium. * $p < 0.05$; *** $p < 0.001$.

Table S1. Sequence of oligonucleotides used as Real-time PCR primers.

Gene	Primers
β-Actin	Fw 5′-TGTGATGGTGGGAATGGGTCAGAA-3′<br>Rv 5′- GCTTCTCTTTGATGTCACGCACGATT-3′
Fsh-R	Fw 5′-ACAGGGTCTTCCTCTGCCAA -3′<br>Rv 5′-TTTCTCCAGGTCCCCAAATCC -3′
Insl3	Fw 5′-CACGCAGCCTGTGGAGAC -3′<br>Rv 5′-GAGAAGCCTGGAGAGGAAGC -3′
3β-Hsd1	Fw 5′-CTCAGTTCTTAGGCTTCAGCAATTAC -3′<br>Rv 5′-CCAAAGGCAGGATATGATTTAGGA -3′
